# Circulatory Collapse due to Hyperinflation in a Patient with Tracheobronchomalacia: A Case Report and Brief Review

**DOI:** 10.1155/2019/2921819

**Published:** 2019-01-29

**Authors:** Niclas Lundström, Gert Henriksson, Ola Börjesson, Malin Jonsson Fagerlund, Johan Petersson

**Affiliations:** ^1^Function Perioperative Medicine and Intensive Care (PMI), Karolinska University Hospital, Sweden; ^2^Department of ENT Surgery, Karolinska University Hospital, Sweden; ^3^Department of Rheumatology, Karolinska University Hospital, Sweden

## Abstract

We present a case of repeated cardiac arrests derived from dynamic hyperinflation in a patient with severe tracheobronchomalacia. Mechanical ventilation led to auto-PEEP with hemodynamic impairment and pulseless electric activity. Adjusted ventilation settings, deep sedation, and muscle paralysis followed by acute stenting of the affected collapsing airways restored ventilation and prevented recurrent circulatory collapse. We briefly review the pathophysiology and treatment options in patients with dynamic hyperinflation.

## 1. Introduction

Patients with expiratory flow limitation are at risk of developing dynamic hyperinflation and auto-PEEP, which in extreme cases can cause circulatory collapse [1]. Aggravated by overzealous positive pressure ventilation, it is crucial that the cause of collapse is immediately recognized in order to relieve intrathoracic pressure and adapt ventilator treatment [2]. Previously described in patients with severe asthma and COPD [3–5], this case also demonstrates the risk of hyperinflation causing cardiac arrest in patients with tracheobronchomalacia. In such a case, rapid stenting of the collapsing airways can be a lifesaving procedure. Patient informed consent was obtained.

## 2. Case

A 66-year-old man arrived at the emergency room after a cardiac arrest with successful prehospital resuscitation.

He had a medical history of hypertension, diabetes, and obstructive sleep apnea. Recently he had also been diagnosed with granulomatosis polyangiitis (GPA) with positive C-ANCA and respiratory tract involvement. Severe tracheobronchial inflammation had led to stenosis and secondary tracheobronchomalacia, with the distal trachea and main bronchi most affected on previous CT scans. At the time of the reported events, he received treatment with azathioprine 100 mg per day and prednisolone 10 mg per day. Noninvasive mask ventilation with positive airway pressure was used at night because of worsened symptoms when recumbent. There was a plan to consult ENT surgeons regarding the possibility of placing airway stents to treat the condition.

Pulmonary function test done the month before showed marked nonreversible expiratory flow limitation with forced expiratory volume in 1 second (0.6 L, 18% of expected) and hyperinflation with a reduced forced vital capacity (2.7L, 58% of expected), an increased residual volume (3.0L, 139% of expected), and functional residual capacity (4.3L, 131% of expected). Total lung capacity and diffusion capacity were normal.

The patient had undergone bronchoscopy under general anesthesia the previous year which was complicated by severe bronchospasm, hypoventilation, and subsequent hypercapnia requiring unplanned delayed extubation and ICU admission.

The cardiac arrest took place during a visit at an out-of-hospital urology clinic. It was preceded by obstructive breathing and coughing leading up to respiratory arrest, and he became pulseless before the arrival of paramedics. Cardiopulmonary resuscitation (CPR) was started. When paramedics arrived, they found pulseless electrical activity, CPR was continued including administration of adrenaline, and after 10 minutes there was return of spontaneous circulation and breathing.

**Figure 1:**
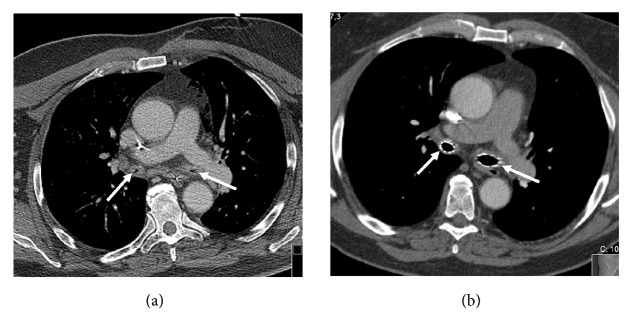
CT imaging before (a) and after (b) stenting. Arrows pointing at main bronchi.

At the hospital emergency room he was unresponsive but with stable pulse and blood pressure. After intubation he was taken to the ICU. Arterial blood gas showed respiratory acidosis with pH at 6.88, PaCO_2_ 16.6 kPa. Ventilating the patient sufficiently to normalize PaCO_2_ was difficult. Regardless of ventilation mode, high inspiratory and end-expiratory pressures were needed for acceptable tidal volumes and gas exchange. PEEP at 14-16 cmH2O was considered optimal. Remaining air flow at end-expiration indicated auto-PEEP; therefore, the expiratory phase was prolonged with I:E ratio 1:4. Bronchoscopy showed very narrow bronchi and inflammation. CT scan confirmed narrow conditions in the proximal airways, especially in the main bronchi (Figure 1(a)). No signs of pulmonary embolism were seen.

The second day at the ICU, the situation quickly deteriorated, with increasing respiratory acidosis. The patient seemed stressed and hypertensive and triggered the ventilator. Suddenly the blood pressure dropped to immeasurable. CRP was started, the ventilator was disconnected, and bag ventilation was attempted. Adrenaline was administered and after about 2-3 minutes of CPR circulation returned. During the CPR, muscle relaxant with rocuronium (Esmeron®) was also administered to ease continued ventilation after return of circulation. The assessment at this point was that hyperinflation and auto-PEEP due to the expiratory flow obstruction likely had caused the circulatory collapse. Increasing sedation and muscle paralysis stabilized the situation and the patient was therefore kept deeply sedated.

The ENT surgeons were consulted to discuss urgent stenting of the collapsing airways. Stenting of each main bronchus using two stents was conducted a few hours later in the operating theater. The procedure was done with aid of rigid bronchoscopy with ongoing jet ventilation during general anesthesia. The diameter of the right main bronchus was approximately 2-3 mm wide and in the left main bronchus hardly any lumen was seen.

With the stents in place (Figure 1(b)), respiratory compliance improved immediately, and peak pressure and PEEP could be reduced with maintained minute volume. The patient was extubated in the operation room. He was neurologically intact and the continued care was uneventful. The following years though, he has needed treatment at hospital several times, due to recurrent respiratory problems with mucus stagnation, infections and granulation tissue in proximity to the stents, well-known complications to long term stent treatment. However, he has experienced no further episode of circulatory collapse.

## 3. Discussion

This case illustrates the risk of dynamic hyperinflation and circulatory collapse in patients with severe expiratory flow limitation and the potentially lifesaving effects of timely airway stenting in patients with obstruction of the central airways as in tracheobronchomalacia. In the following, we briefly discuss lung hyperinflation, auto-PEEP and circulatory compromise and lastly tracheobronchomalacia.

**Figure 2:**
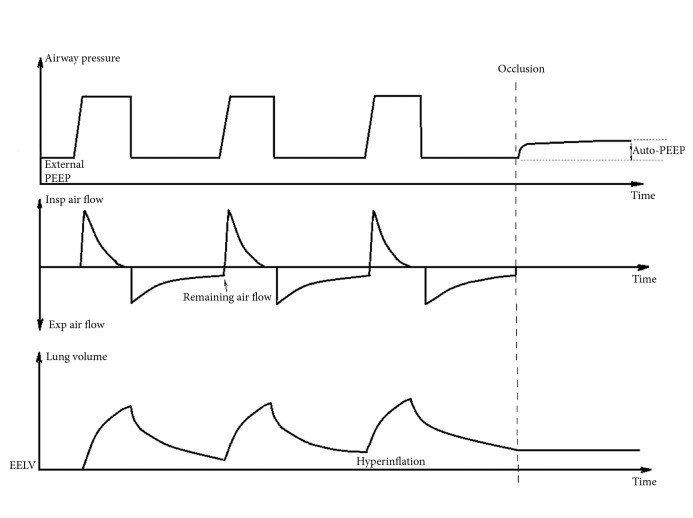
Ventilatory curves for three consecutive breaths during pressure controlled ventilation in a patient with dynamic hyperinflation and auto-PEEP, measurement of static auto-PEEP after occlusion.

Lung hyperinflation essentially means that the lung volume at the end of expiration is above normal [6, 7]. Static hyperinflation refers to a consistent increase in the functional residual capacity (FRC). Dynamic hyperinflation refers to a variable increase in end-expiratory lung volume (EELV), often caused by expiratory flow limitation that prevents the lung from emptying to its resting volume before inspiration. Dynamic hyperinflation varies with changes in flow limitation, minute ventilation, and breathing pattern. In this situation of incomplete expiration, alveolar pressure at end-expiration remains higher than proximal airway pressure at the start of next inspiration. This is called intrinsic PEEP or auto-PEEP [1]. Physiologically, auto-PEEP differs from externally applied PEEP set on a ventilator, as auto-PEEP is present only distal to airways with flow limitation and not equally distributed in the lungs [2, 8]. During invasive ventilation auto-PEEP leads to decreased lung compliance, resulting in reduced tidal volumes during pressure controlled ventilation and increased peak pressures in volume controlled modes [9]. Auto-PEEP can be measured in most ventilators from the airway pressure during prolonged expiration with a closed expiration valve (Figure 2). This requires a passive patient.

Auto-PEEP can generate the same hemodynamic effects as externally added PEEP. The resulting elevated intrathoracic pressure reduces venous return and thus cardiac output [10–13]. Hyperinflation may also reduce cardiac output through increased pulmonary vascular resistance, impairing right ventricular function leading to reduction of left ventricular preload. Bradycardia and vasodilation via autonomic reflexes are also possible adverse effects of hyperinflation [14].

As described, our patient suffered two episodes of cardiac arrest, and our discussion centers around the second occasion during invasive ventilation. However, in patients with obstructive lung disease, a worsening of expiratory flow limitation and increased breathing frequency can cause significant dynamic hyperinflation and auto-PEEP also during spontaneous breathing. This can both increase work of breathing further and impair cardiac output [6]. As the primary cardiac arrest was preceded by obstructive respiratory distress with forced and rapid breathing, we believe increasing dynamic hyperinflation and auto-PEEP played a key role in the rapid development of respiratory failure and circulatory compromise with hypoxemia and hypotension leading to cardiac arrest.

**Figure 3:**
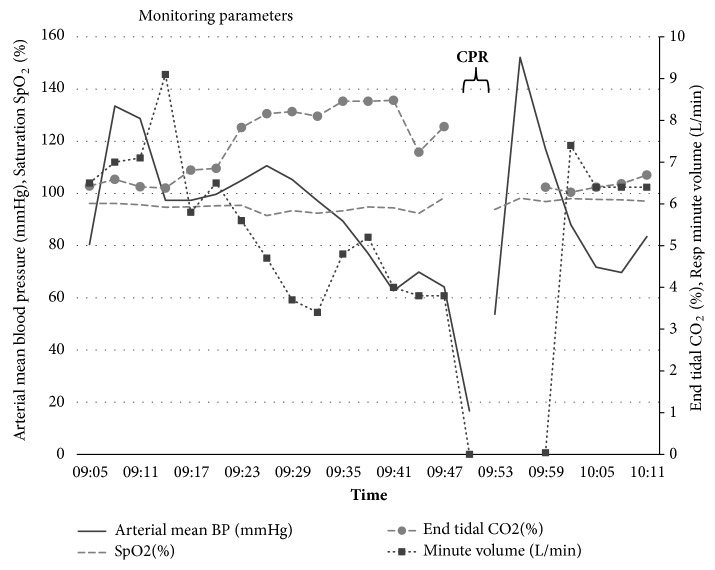
Vital parameters. Preceding the second cardiac arrest, expired minute volume is reduced, resulting in elevated end tidal carbon dioxide concentration. Blood pressure is initially elevated, probably secondary to stress, before it suddenly drops. Saturation is maintained through increased oxygen fraction in inspired air.

The second cardiac arrest during invasive ventilation was preceded by acceptable oxygenation but ventilation difficulties with reduced tidal volumes and hypercapnia. After a brief period of hypertension, blood pressure dropped quickly before onset of the cardiac arrest (Figure 3). The circulatory collapse can in this case be considered caused directly by dynamic hyperinflation and deleterious auto-PEEP levels generated during positive pressure ventilation.

The sudden collapse could have been related to swelling of the airways with further lumen reduction, and stress from light sedation with patient triggered breaths and forced expiration likely worsened airway collapse and flow limitation further. With diminishing expiration, auto-PEEP approaches the inspiratory pressure delivered by the ventilator. Progressively increasing respiratory acidosis and respiratory efforts sets the stage for a vicious circle culminating in extreme intrathoracic pressures and circulatory collapse. Attempting to increase the minute ventilation with the ventilator in this situation could aggravate the situation even more by further increasing auto-PEEP. Instead tidal volume or respiratory rate needs to be reduced, with permissive hypercapnia if necessary, to allow lung emptying and reduce auto-PEEP. This might require heavy sedation to suppress the patient's ventilatory drive. If sedation is insufficient to achieve patient-ventilator synchrony or is poorly tolerated due to hemodynamic instability, muscle paralysis might be necessary [2, 8, 9].

If the situation progresses to cardiac arrest, decreasing intrathoracic pressure by disconnecting the patient from the ventilator might restore venous return. Switching to manual bag ventilation is crucial to allow sufficient expiration time as rapid bag ventilation may also generate high auto-PEEP which could impair CPR outcome [2, 15]. Hyperinflation and auto-PEEP have been suggested as underestimated cause of persistent pulseless electrical activity during CPR in patients with obstructive airway diseases [5, 16].

Any action that might reduce flow limitation, e.g., bronchodilating therapy or exchange of an obstructed endotracheal tube, should be taken as rapidly as possible. The placement of stents to improve airway patency of the most affected bronchi solved the most severe expiratory air flow limitation in our case, preventing further episodes of dynamic hyperinflation and circulatory compromise which we believe confirms the pathophysiology outlined above.

Applying external PEEP may be useful in patients with flow limitation and dynamic hyperinflation, especially in cases with expiratory airway collapse such as COPD or tracheobronchomalacia. It may improve the airway patency, equalize PEEP between lung regions, and improve gas exchange. As long as applied PEEP is lower than auto-PEEP, it should exert little effect on peak pressure. In pressure support mode or noninvasive ventilation, external PEEP can ease triggering and reduce work of breathing. With applied PEEP balancing auto-PEEP, the relative negative pressure change needed for inspiration is reduced. Some literature recommended applying external PEEP at 75-85% of estimated auto-PEEP [8–10].

## 4. Tracheobronchomalacia

Our patient was diagnosed with GPA, a disease commonly involving the lower respiratory tract, e.g., causing subglottic stenosis, alveolar bleeding, pulmonary granulomas, tracheobronchial inflammation, and tracheobronchomalacia [17, 18].

Tracheobronchomalacia is a condition with weakened tracheal or bronchial cartilage. It can be secondary to trauma, surgery, intrathoracic processes, chronic inflammation, and necrosis in the bronchial tissues. Obstruction of air flow primarily occurs at active expiration, when the intrathoracic pressure exceeds the intrabronchial pressure, leading to collapse of the weakened parts [19, 20].

**Figure 4:**
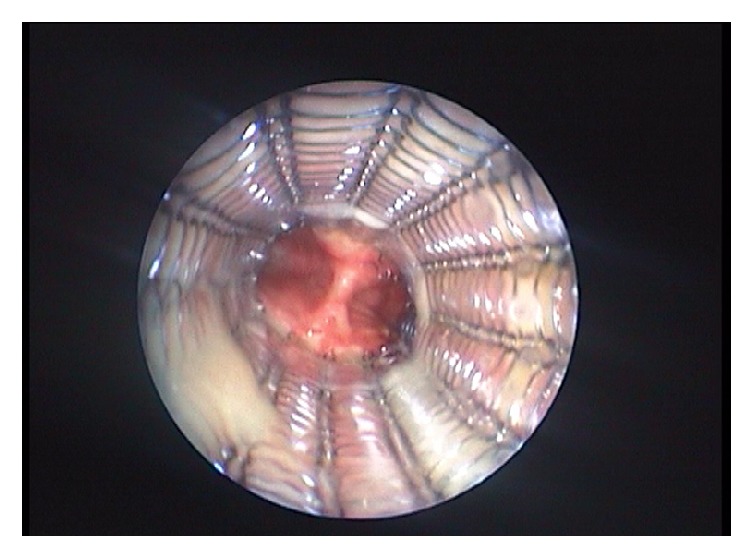
A metal stent in the trachea. This nitinol stent is covered except at the ends. The image is from another patient.

If medical treatment is insufficient, further treatment include laser treatment, surgery, and stenting with silicone or metal stents (Figure 4) in order to prevent airway collapse [18, 19]. Stents are ideally inserted for a shorter time, but sometimes long term treatment is necessary, which can cause problems with mucus stagnation, infections, and granulation tissue forming at the ends of the stent. Surgical tracheobronchoplasty is done at some centra, a procedure that aims to stabilize the airway by suturing a mesh to the posterior membrane of the trachea and bronchi. [20–22].

## Additional Points

*Key Points.* This case highlights potentially life-threatening effects of dynamic hyperinflation and auto-PEEP caused by flow limitation in patients with tracheobronchomalacia and the difficulties with mechanical ventilation in these patients. Acute stenting of the affected airways is an important treatment option.
